# Cutaneous Wound Healing: A Review about Innate Immune Response and Current Therapeutic Applications

**DOI:** 10.1155/2022/5344085

**Published:** 2022-04-25

**Authors:** Yara Adib, Armand Bensussan, Laurence Michel

**Affiliations:** ^1^INSERM U976, Hôpital Saint-Louis, Paris, France; ^2^Université de Paris, Paris, France; ^3^Laboratoires Brothier, Nanterre, France

## Abstract

Skin wounds and compromised wound healing are major concerns for the public. Although skin wound healing has been studied for decades, the molecular and cellular mechanisms behind the process are still not completely clear. The systemic responses to trauma involve the body's inflammatory and immunomodulatory cellular and humoral networks. Studies over the years provided essential insights into a complex and dynamic immunity during the cutaneous wound healing process. This review will focus on innate cell populations involved in the initial phase of this orchestrated process, including innate cells from both the skin and the immune system.

## 1. Introduction

Wound healing is a highly regulated physiological process that involves interactions between resident cells, infiltrating cell subtypes, extracellular matrix molecules, and cytokines. The consecutive steps of the healing process tend to achieve both control of the external aggression, eradication of the eventual foreign adversary, and homeostasis, in order to guarantee the maintenance of tissue integrity and function of wounded tissue after trauma with a complete final tissue regeneration [1, 2]. By definition, a wound is a damage or disruption to the normal anatomical structure and function of the tissue. It can range from a simple break in the epithelial integrity of the skin to a more profound lesion reaching the dermis or extending into subcutaneous tissue with damage to other structures such as muscles, vessels, and organs (lungs, intestine, and cornea) [3]. It has been demonstrated that despite many differences among organs and injuries, the wound healing of the skin or internal organs, like the heart, is characterized by a similar, complex series of overlapping events involving multiple different cell types and cellular interactions [4]. This review will overview the major cutaneous wound healing events and focus on innate cells from both the skin and the immune system. Some examples might also be quoted from other types of wound healing due to the similarity between mechanisms.

The skin as the external envelope of the body serves as a physical barrier, mainly by the structure of the stratum corneum that assures the primary defense against environmental, physical aggressions, and external pathogen invasion. Additionally, superficial skin layers are colonized by populations of microorganisms that form the cutaneous commensal microbiota, participating in the instruction and support of the skin immune system [5–7]. A secondary defense line is conducted by innate immune cells (mast cells, neutrophils, macrophages, and innate lymphocyte cells), by resident dendritic and Langerhans cells that link innate and adaptive immunity, and by nonimmune cells like epidermal keratinocytes and melanocytes, as reviewed by Rodrigues et al. [8]. Cells from the adaptive immune system (T lymphocytes; cytotoxic T cells, helper T cells, and *γδ* T cells) participate later in the skin defense and its immune activity [9]. All these elements play a central role in orchestrating the tissue healing process and are actively engaged in reestablishing homeostasis after tissue injury through multiple mechanisms [10]. The main resident or recruited innate cells are listed in Table 1.

## 2. Cutaneous Wound Healing Stages

**Figure 1:**
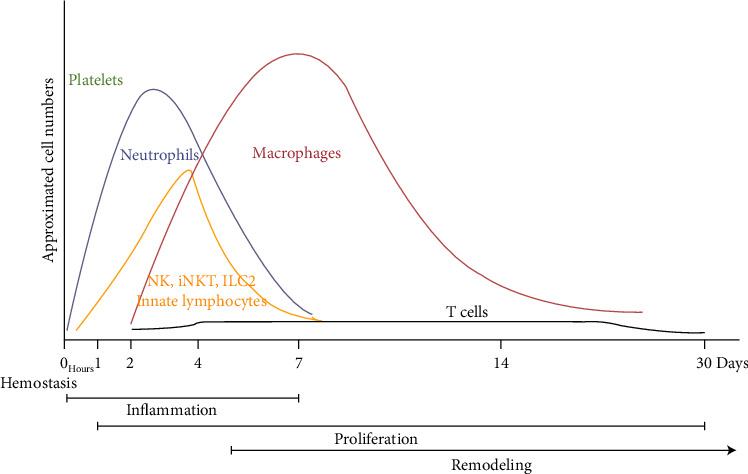
Wound healing process. Timeline of immune cell migration in relation with the phases of wound healing (adapted from [26]).

Temporally, cutaneous wound healing involves four consecutive stages (Figure 1): 1—hemostasis (within minutes to hours), 2—inflammation (1-7 days), 3—the resolution of inflammation overlaps with the beginning of the third phase, which is the proliferative process of repair (weeks to months), and 4—the remodeling phase starting about three weeks postinjury and maintained up to 2 years [25].

Dermal skin lesions provoke rupture of blood vessels leading to bleeding and subsequent rapid accumulation of platelets and thrombogenesis, initiating the clotting process, where a successive conversion of clotting enzymes into their active forms leads to a fibrin clot [27]. In this first step of the wound healing process, aggregated platelets degranulate and release growth factors and chemotactic factors such as tumor growth factor (TGF-*β*1), platelet-derived growth factors (PDGFs), and platelet factor 4 (PF4) [28].

Under the local release of these chemotactic factors, the inflammatory phase begins to take place with the diapedesis of circulating innate immune cells such as neutrophils and macrophages through the intact walls of the capillaries [29]. These cells will interact with the extracellular matrix (ECM) through the integrin-binding site to migrate towards the wound bed [30]. To note, the quality and duration of the inflammatory response define the progress of the healing wound. If inflammation persists for an extended period of time, wound healing will be impaired, and chronic ulcers might be generated [31].

The third phase of the wound healing process concerns, besides the resolution of the inflammatory phase, the proliferative phase, during which the tissue begins to heal by the effective closure of the wound, obtained by the migration of keratinocytes that will cover the lesion [32]. Dermal fibroblasts participate in this closure phase by their migration, their local differentiation into myofibroblasts, and the production of a new ECM [33, 34]. The key events during this phase are the regeneration of a novel ECM, the formation of a new epithelial barrier (i.e., reepithelialization), the establishment of sufficient blood supply through angiogenesis, and strengthening of the injured dermal tissue (i.e., fibroplasia).

The fourth and final stage is known as the remodeling process, with as main characteristics, the change of ECM composition [35]. This phase helps to generate great tensile strength with a gradual turnover of collagen as type III collagen undergoes degradation and type I collagen synthesis increases. The balance between the activities of matrix metalloproteinases (MMPs) and tissue inhibitors of metalloproteinases is noteworthy critical to wound repair and remodeling [36].

## 3. Host Innate Response

Different signals can induce the activation of resident innate cells and the recruitment of circulating inflammatory cells into the wound site; they include the release of alarmins, also known as damage-associated molecular patterns or DAMPs by damaged host cells (e.g., uric acid, DNA, RNA, and extracellular matrix components) [37, 38], the release of inflammatory mediators by platelets during the hemostasis phase (e.g., PF4 and CXCL8) [39], and the production of reactive oxygen species (ROS) by many immune and nonimmune cells [40]. Indeed, ROS (e.g., O^·−2^, OH^·^, and H_2_O_2_) play a key immediate-early role through platelet activation, leukocyte recruitment, and keratinocyte and fibroblast proliferation and migration [40, 41]. The innate cells present at the injury site (keratinocytes, Langerhans cells, mast cells, and macrophages) recognize potential pathogens through multiple pattern recognition receptors (PRRs), including toll-like receptors (TLRs) that bind to pathogen-associated molecular patterns (PAMPs) associated with microbes [42]. This binding triggers signaling events that activate antimicrobial defense systems and stimulate proinflammatory cytokine production by the inflammatory cells [43].

**Figure 2:**
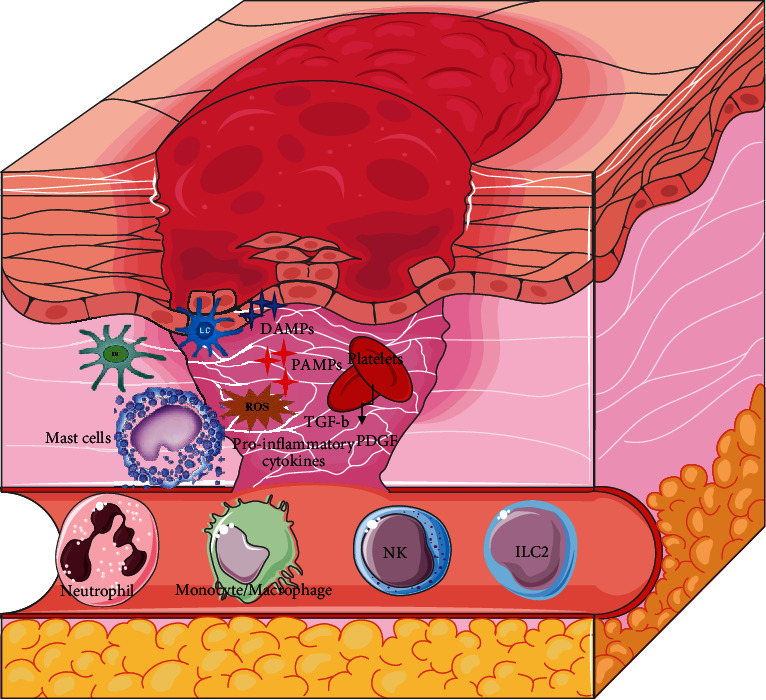
Inflammatory phase of wound healing. Main innate cells that invade wound bed in response to local stimuli upon injury.

As introduced, various immune cell types are mobilized following tissue injury (Figure 2), and several cell subsets contribute to the production of cytokines and growth factors during normal and impaired wound healing. Indeed, there are a large variety of crucial mediators for wound healing and the inflammatory process in both the innate and adaptive arms of the immune system. Among them, the ability of the innate immune cells to communicate with epithelial cells for an effective immune response is a key feature of the cutaneous immune system.

Herein, we put the light on the early steps of the wound healing process and focus on innate cell subtypes that are involved, including skin resident cells, and recruited circulating ones: 1—keratinocytes (KCs), 2—Langerhans cells (LCs) and dermal dendritic cells (DCs), 3—mast cells, 4—neutrophils, 5—monocytes/macrophages, and 6—innate lymphoid cells (ILCs). However, it must be underlined that a fragile balance exists between a normal and an excessive inflammatory response. In some cases, the sustained presence of inflammatory cells, such as neutrophils or proinflammatory macrophages producing inflammatory mediators locally, will induce the persistence of chronic wounds [44]. Therefore, controlling/modifying the immune system has become of major interest for immunotherapy to promote tissue repair and regeneration.

## 4. The Contribution of Resident Innate Skin Cells in Cutaneous Wound Healing

### 4.1. Keratinocytes (KCs)

Keratinocytes are the major cellular components of the epidermis, involved in both the physical and immune defense of the host [45]. They act as sentinels by sensing microbial pathogens or physical insults [46]. Upon injury, KCs release mediators and consequently participate in the activation of cutaneous immune cells, such as mast cells, dendritic cells, and Langerhans cells, and in the recruitment of circulating innate immune cells, including neutrophils and macrophages, to the wound site [47]. The numerous mediators produced by KCs include cytokines (IL-1, TNF-*α*, IL-6, and IL-10), chemokines (CXCL-8, CXCL1), growth factors (TGF-*β*, GM-CSF, PDGF, and VEGF), and antimicrobial peptides (AMPs), such as *β*-defensins 2, 3, 4, cathelicidins, and S100 family members [11]. AMPs are peptides constituted by 12 to 50 amino acids with an amphipathic structure and are well described for their antimicrobial activity [48]. Natural or synthesized AMPs are used to treat bacterial infections [49] and modulate the inflammatory immune response during wound healing [50]. As an example, a small designed peptide tiger 17 was shown to modulate several events of wound healing in a murine model of full-thickness wounds [51]. In this model, it favors the induction of the recruitment of macrophages to the wound site and induces the stimulation of TGF-*β* secretion by fibroblasts as well as the promotion of the migration and proliferation of keratinocytes and fibroblasts [51]. Other AMPS like SR0379 and epinecidin-1 have been shown to be involved in collagen synthesis by fibroblasts during the remodeling phase [52].

In addition to AMPs, keratins released by KCs could act as alarmins upon injury: the expression of KRT6, KRT16, and KRT17 by stressed keratinocytes at the suprabasal layers of the epidermis represents a highly activated and proliferative stage of these cells under pathological conditions [53]. These keratins contribute to wound repair by properly regulating the production of innate danger signals [54] and by optimizing several keratinocyte functions, such as cell adhesiveness, mechanical integrity/resilience against physical stress, and proliferative potential [55].

Of interest, the use of keratin-based wound dressings offers a novel approach to wound management. As an example, the potent role of keratin in diabetic wound healing has been studied by using biocompatible and biodegradable keratin-based wound dressings “*fur keratin-derived powder* (FKDP)” on full-thickness wounds in diabetic mice [56]. Antimicrobial efficiency and a faster healing process were observed in FKDP-treated wounds compared to untreated wounds.

### 4.2. Langerhans (LCs) and Dermal Dendritic Cells (DCs)

Langerhans cells represent 2 to 4% of the epidermal cell population and are dendritic cells sharing typical features with DCs, especially in terms of migratory potential and ability to stimulate T cells [57]. In response to trauma, LCs extend their dendrites vertically through epidermal tight junctions and engulf foreign antigens via dendrite tips [58]. Upon antigen recognition, LCs downregulate their E-cadherin expression (this expression normally affording for their contact with keratinocytes) [59] and are able to migrate through the dermoepidermal junction throughout degraded ECM under the action of locally released MMP2 and MMP9 [60]. Chemokines secreted in the wound site guide their migration from the epidermis through the dermis into the draining lymph nodes, where LCs initiate a T cell-mediated adaptive response [61, 62]. Together with dermal DCs, as discussed thereafter, they are the major antigen-presenting cell (APC) subsets responsible for initiating immune responses in the skin. In addition to their immunogenic role, they display marked functional plasticity [63], serving as tolerogenic cells by their increased capacity to produce interleukin-10 (IL-10) that induces the activation and proliferation of skin resident T regulatory cells [64].

In inflamed or injured skin, endogenous alarmins and cytokines produced by nearby KCs, such as monocyte chemoattractant protein-1 (MCP-1), as well as other inflammatory signals like PAMPs, can promote the recruitment, activation, and maturation of LCs, as shown by their increased T cell-stimulatory capacity and increased MHC-II molecule expression [65]. A beneficial role for human LCs in cutaneous wound healing has been highlighted in chronic wounds [66]. In particular, in diabetic foot ulcers, LCs were present in high numbers and associated with a better healing outcome [67].

Dermal DCs represent a complex heterogeneous population, classified into conventional DCs or nonconventional DCs (plasmacytoid DCs) that differ in ontology and specific functions, as reviewed by Balan et al. [68]. As their epidermal counterpart, Langerhans cells, the primary function of dermal DCs is to deliver antigen to CD8 and CD4 T cells, playing a crucial role in the immune system by linking the innate and the adaptive immunity [69]. Evidence about the role of dermal DC in wound closure has been demonstrated by the crosstalk between epithelial cells and DCs residing in the corneal epithelium during corneal epithelial wound healing; the consequences of this interaction on wound healing have been studied using B6-diphtheria toxin receptor transgenic mice (B6-DTR) depleted of their DC subtype [70]. In DT-injected corneas, impaired wound closure and attenuated wound-induced expression of CXCL10, thymic stromal lymphopoietin (TSLP), IL-1, and IL-1Ra, produced by migratory epithelia, were observed compared with PBS-injected ones.

These results identified an additional function of DCs in interacting with adjacent epithelial cells for maintaining tissue homeostasis and for tissue repair. In the case of burns, the destroyed epidermis and dermis induce a great susceptibility of wounds to infections [71]. Using CD11c-DTR transgenic mice, Vanish et al. showed a significantly decreased wound closure and granulation tissue formation in DC-depleted mice compared to control mice [72]. This study suggests that dermal DCs present in the regenerating dermis at 4 days postburn participate in accelerating wound healing by enhancing fibroblast proliferation and production of TGF-*β* without leading to excessive collagen deposition and scar formation.

Of note, both Langerhans cells and a subpopulation of dermal dendritic cells express langerin, a C-type lectin receptor [73]. The ablation of langerin-positive cells in mice (a langerin-DTR depletable mouse model) induced the healing of a full-thickness excision wound by increased neo-epidermis and granulation tissue formation [74].

Altogether, these recent data demonstrate the involvement of Langerhans cells and dendritic cells in early phases of wound healing, thus linking innate and adaptive immunity.

### 4.3. Mast Cells (MCs)

**Figure 3:**
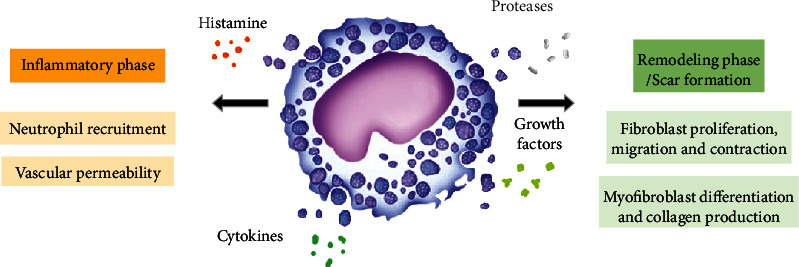
Mast cell activities during wound healing. Secretion of mediators by MCs affects several phases of wound healing. As detailed here, MCs stimulate inflammation by releasing proinflammatory mediators inducing vascular permeability and recruitment of neutrophils (left side). MCs influence the remodeling phase and scar formation by secreting proteases that cleave extracellular matrix components and by producing a variety of mediators that stimulate fibroblasts (right side).

Mast cells derived from the myeloid stem cells are key effector cells of the innate immune. They are abundant in barrier organs such as the skin, representing 2 to 8% of dermal cells [75]. Since their discovery in the late 1800s, they have been intensively studied as major actors in allergic inflammation. Although they are one of the first cells to respond to injury, their precise role in wound healing remains debated. Following injury, they accumulate in the wound bed within the first 24 hours, in correlation with the level of MCP-1 released by resident KCs and macrophages [76]. Moreover, even when neutrophils and lymphocytes disappear, few resident MCs and macrophages are still present during the remodeling phase, and this suggests that mast cells may participate in all stages of wound healing [77]. In response to wounding, they release their cytoplasmic granules containing histamine, serotonin, chymase, and tryptase, and they produce various cytokines and inflammatory mediators, including tumor necrosis factor (TNF-*α*), IL-1, and growth factors, such as TGF-*β*1 or PDGF [78]. Secreted histamine and vascular endothelial growth factor (VEGF) stimulate vessel permeabilization, promoting the influx of neutrophils, macrophages, and additional MCs into the tissue (Figure 3). In addition, cytokines released by MCs also promote proinflammatory mediator production by resident cells that will favor the recruitment of additional circulating immune cells to the wound site and the activation and proliferation of endothelial cells for the revascularization of injured tissue [79, 80]. However, cellular interactions between mast cells and fibroblasts have shown that histamine, TGF-*β*, and some serine proteases released by MCs induce proliferation and migration of fibroblasts in normal skin, and this might promote fibrotic responses [81].

In accordance, the involvement of mast cells in the process of wound healing has been studied using mast cell-deficient mice such as WBB6F_1_-*Kit^W/W-v^* and C57BL/6-*Kit^W-sh/W-sh^* mice [82]. In MC-deficient Kit^W^/Kit^W-v^ model, wound closure was significantly impaired in the absence of MCs during the first 6 days of wound healing with impaired extravasation and recruitment of neutrophils to the wounded areas [83]. To validate the effective involvement of MCs, an adoptive transfer of functional MCs to Kit^W^/Kit^W-v^ mice led to a complete normalization of wound closure, restored extravasation, and neutrophil accumulation.

Another role that might be important during the process of wound healing is the MC contribution to antibacterial defense by releasing antimicrobial peptides [84] and forming extracellular traps (ETs) [85], as recently reviewed by Elieh Ali Komi et al. 2021 [86]. This process of defense against infection has been first described in neutrophils under the term Neutrophil Extracellular Traps (NETs), corresponding to the NETosis mechanism [87]. NETosis has been shown to play a potent role in wound healing [88]. Such process of defense is also used by macrophages in response to various stimuli, known as Macrophage Extracellular Traps (osis) (METosis) [89]. These ETs are structures composed of granular and nuclear constituents that disarm and kill bacteria extracellularly [87]. However, excess or deregulation of these processes, particularly NETosis, can cause tissue damage and delayed wound healing by amplifying inflammation [90]. MCs are also indirectly involved in the defense against bacterial infections by releasing soluble factors that recruit or activate immune cells, such as neutrophils, dendritic cells, and T cells. Zimmerman et al. [91] studied the antibacterial role of mast cells in wounds infected with Pseudomonas aeruginosa of either WT mice or MC-deficient Kit^W^/Kit^W-v^ mice. In contrast to WT mice, MC-deficient mice exhibited an impaired skin wound healing in response to this infection that was restored after local adoptive transfer of bone marrow-derived cultured MCs (BMCMCs). However, the count of the numbers of neutrophils was not different in MC-deficient mice and WT mice. The results of Zimmerman's study brought evidence of a potent antibacterial effect of MCs, independently of neutrophils, in infected wounds; this was linked to mast cell-derived IL-6 that led to the release of antibacterial AMPs by keratinocytes and turned these latter into better bacterial killers [91].

However, through their production of numerous proinflammatory mediators, MCs may alter the wound remodeling process, as shown by the promotion of chronic nonhealing ulcerations associated with excess degranulation and increased numbers of skin MCs [92]. Some studies also suggested a role of MCs in fibrotic conditions, where their increased number was associated with an excessive production of chymase and tryptase (Figure 3). Indeed, these enzymes have been shown to stimulate fibroblast proliferation and myofibroblast differentiation via the TGF-*β*1/Smads signaling pathway leading to the synthesis upregulation of collagen I, collagen III, and other extracellular matrix components, as observed in hypertrophic scars [93–95] and keloids [77, 96].

Although wound healing in rodents is fundamentally different from that of humans and occurs via tissue contraction, some murine models overcome this difference by incorporating a splint around the wound, enabling the repair process to become dependent on epithelialization, cellular proliferation, and angiogenesis, which closely mirror the biological processes of human wound healing [97, 98]. The role of mast cells in wound healing and scar formation was confirmed by using three different genetically mast cell-deficient murine models that mimic the physiological repair of cutaneous wounds in humans [99]. Wound closure kinetics were studied in splinted wounds; the results did not bring any evidence of differences in the time of wound closure, the wound size, quantity of collagen, and collagen microarchitecture between these models and the control mice, suggesting that MCs are not required for wound healing in these murine models.

However, the use of mast cell inhibitors such as disodium cromoglycate in mouse models reduces scar formation and the production of proinflammatory cytokines like IL-1*β* and CXCL1 without affecting the reepithelialization of the wound or further weakening the healed wound [100]. The modulation of mast cell activity by ketotifen or sodium cromoglycate has also been studied in Yorkshire pigs that exhibit a wound healing process close to the human one or in red Duroc pigs that form pathogenic fibroproliferative or hypercontractile scars [101]. This study highlighted a major role of mast cells in preventing wound contraction and a slight requirement in healing cutaneous wounds.

Altogether the involvement of mast cells in cutaneous wound healing has been clearly demonstrated during the last two decades, although these cells might favor scar fibrosis in some pathological cases, such as keloids, by the production of active substances.

## 5. The Contribution of Innate Immune Cells in Cutaneous Wound Healing

### 5.1. Neutrophils

Wound healing is a dynamic process that involves not only resident mast cells but also infiltrating neutrophils and macrophages. Neutrophils appear shortly after injury; their numbers reach a maximum level between day 1 and day 2, followed by the infiltration of monocytes into the wound in day 2 to day 3 postinjury [102–104]. Neutrophils and monocytes begin to emigrate from blood capillaries into the wounded tissue in response to proinflammatory cytokines and chemokines present in the wound bed during the hemostasis phase. These include CXCL8 and leukotriene B4 (LTB4), two strong inducers of neutrophil chemotaxis [105, 106]. Neutrophils locally phagocytose and digest bacteria, pathogens, and tissue debris using a variety of antimicrobial substances, such as ROS, cationic peptides, and proteases (elastase, cathepsin G, proteinase 3, and urokinase-type plasminogen activator) [16, 107]. As introduced above, they also exert antibacterial defense by NETosis [87]. In addition, they release cytokines, including IL-1*α* and *β* and TNF-*α*, which provide some of the earliest signals that activate local fibroblasts and keratinocytes [15]. Very recent studies have distinguished two distinct subsets of neutrophils in the context of the myocardial infarction: N1 that are proinflammatory and antitumoral cells, characterized by a higher level of intercellular adhesion molecule- (ICAM-) 1 expression and high secretion of IL-12, CCL3, and IFN-*γ*-induced protein 10 (IP-10)/ CXCL10; N2 that exhibit anti-inflammatory and protumoral characteristics, with a high cell surface expression of C-X-C motif chemokine receptor 2 (CXCR2) and high secretion levels of IL-8, IL-10, and CCL2 [108, 109]. The anti-inflammatory N2 subset might play a potent role in tissue regeneration, although the short life of this N2 subset did not enable a clear demonstration of their role in wound healing and tissue regeneration [110]. Further studies are needed to clearly demonstrate the functions and phenotypic profiles of this N2 subset in wound reparation.

The major role of neutrophils in the wound healing process was demonstrated by using a model of neutrophil knockdown mice obtained by the injection of a specific anti-mouse neutrophil antibody (rat anti-mouse Gr-1 monoclonal antibody RB6-8C5) [111]. Of interest, this study has shown that the process of wound repair was delayed in old mice as compared to young ones, in links with neutrophil dysfunction reported as a feature of immune aging [112, 113].

However, some negative effects of neutrophils in chronic wounds have been also observed. Indeed, since active neutrophils are present for an extended period in chronic wounds, their excessive protease production could cause inactivation of growth factor receptors and the degradation of the extracellular matrix that can enlarge the area in need of repair [114]. This suggests that an enhancement in the healing process of chronic wounds might be obtained by neutralizing neutrophil proteases without losing their capacity to eliminate pathogens and to limit infection. Of interest, the use of local or systemic administration of granulocyte-macrophage colony-stimulating factor (GM-CSF) was shown to increase infiltrating neutrophil count and phagocytosis index simultaneously, enhancing acute and chronic wound healing [115–117].

### 5.2. Monocytes and Macrophages M1/M2

During wound healing, hypoxia is one of the prominent microenvironmental factors in tissue injury. It induces stimulation of various cell populations, including resident macrophages that produce mediators and chemoattractants that enhance leukocyte recruitment. Circulating monocytes enter the wound in tandem with the influx of neutrophils and differentiate into mature tissue macrophages locally [118]. Several wound macrophage subsets could be derived from monocytes, according to the time of recruitment and the local wound environment.

**Figure 4:**
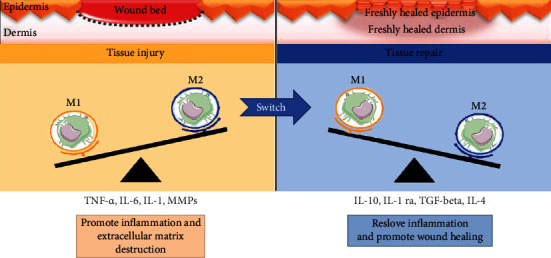
Macrophage polarization (switch M1/M2) during wound healing.

Macrophages commonly exist in two distinct subsets: M1 macrophages, currently named “classically activated” macrophages, characterized by the expression of a combination of cell surface markers (CD80, CD86, TLR2, and TLR4) and the production of proinflammatory key cytokines (TNF-*α*, IL-12, IL-6, IL-8, and IFN-*γ*) and by their phagocytic ability; M2 macrophages called “alternatively activated” macrophages exhibit CD200R, CD206, and CD163 as cell surface markers; they produce IL-10 and TGF-*β* and exert anti-inflammatory functions through these mediators [119]. The switch M1/M2, defined as macrophage polarization, occurs during wound healing [120] (Figure 4). Within 2-4 days, M1 macrophages enter the injury site and produce their proinflammatory mediators in response to bacteria or/and local IFN-*γ* and TNF-*α* present in the injured tissue [121].

The M1 macrophages will phagocyte apoptotic neutrophils that had undergone programmed cell death, a process defined as efferocytosis that enables a progressive attenuation of the local inflammation [122–126]. Besides efferocytosis signaling that regulates macrophage inflammatory responses and favors an anti-inflammatory M2 phenotype that promote tissue repair [125, 126], macrophages also participate in tissue clearance by METosis as previously introduced [89].

Activated M2 macrophages release potent growth factors (PDGF, fibroblast growth factor (FGF), VEGF, TGF-*β*, and TGF-*α*), chemotactic factors (fibronectin), and anti-inflammatory cytokines like IL-10 or IL-4 [127, 128]. By that way, they initiate granulation tissue formation by activating fibroblast proliferation, favoring their migration and ECM production, as well as angiogenesis. Thus, it can be concluded that macrophages through their plasticity play a key role in the transition between inflammation and tissue repair.

In order to define the mechanisms of cutaneous wound healing depending on macrophages or not, a mouse model allowing the depletion of macrophages in a temporally controlled manner was used to target each phase of wound healing one by one, separately [129]. This study validated the involvement of macrophages in all steps of wound healing, confirmed by a delayed wound closure, a decrease in granulation tissue formation and angiogenesis, and a decrease in collagen synthesis due to a reduced level of myofibroblasts under macrophage depletion. It has also been shown that the selective depletion of the anti-inflammatory subset M2 using blockade of the colony-stimulating factor (CSF-1) signaling induced a prolongation of the inflammatory phase in surgical wounds through an increase in the numbers of neutrophils and of M1 macrophages as well as an attenuation of collagen deposition [130]. In another study, the systemic depletion of M2 macrophages, which are present for 3-4 weeks in subacute wounds, reduced hypertrophic scar formation [131].

In order to improve wound repair, cell therapy or targeted therapy affecting the role of macrophages has been assessed for enhancing this process. For instance, the topical application of ex vivo generated M2 macrophage subphenotype was shown to be inefficient in mice, indicating that the manipulation of the wound environment by exogenous administration of M2-polarized macrophages did not bring an efficient therapeutic approach [132]. Furthermore, since humans are consistently exposed to *α*-Gal antigens produced by enteric florae and ingested food (carbohydrate from animal meat consumption), the interaction *α*-Gal antibody : anti-Gal antigen could have excellent potential as a therapeutic strategy. Accordingly, recent studies have highlighted the benefits of using *α*-Gal nanoparticles or liposomes in accelerating healing of impaired wounds in diabetic patients and elderly individuals by the enhancement of macrophage invasion as well as a by privileging M2 tissue reparative phenotype [133, 134]. Another promising therapeutic target might be IL-1*β*, knowing that this cytokine plays a key role in sustaining the proinflammatory macrophage phenotype. Indeed, targeting the IL-1*β* pathway *in vitro* using neutralizing antibodies or *in vivo* using macrophages isolated from IL-1R1 knockout mice helped the improvement of the healing of diabetic wounds [135]. Another pharmacological approach might be the use of dexamethasone, known as a highly potent glucocorticoid routinely used as an anti-inflammatory agent. Liposome formulations for local delivery of dexamethasone to primary human macrophages have been assessed *in vitro* and showed an increased efferocytosis activity and a decreased IL-6 and TNF-*α* production by macrophages [136]. Therefore, dexamethasone might favor an anti-inflammatory/proresolution macrophage phenotype appropriate for tissue repair. These findings and applications came from the increasing knowledge of molecular and cellular mechanisms that help develop and define potential pathways, considering the various underlying pathophysiological factors and different forms of macrophages in wounds and their unique requirements.

### 5.3. Innate Lymphoid Cells

#### 5.3.1. Natural Killer (NK)

The presence of NK cells during the early inflammatory phase of wound healing has been reported in the first 3 days postinjury with a progressive decrease to negligible levels at 7 to 14 days postwounding [137]. Although the direct contribution of NK cells in human cutaneous wound healing remains unclear, their cytotoxic capacity and their immunoregulatory function enable them to control infections and to prevent the development of chronic inflammatory diseases.

Indeed, NK cells are cytotoxic innate immune cells that contain lytic granules (perforin and granzymes) [138] and will degranulate upon activation following injuries and bacterial invasion.

They secrete IFN-*γ* [139], responsible for both neutrophil recruitment and apoptotic cell clearance, as well as for the activation of immune cells such as macrophages [20]. The role of IFN-*γ* in wound healing remains however controversial. On the one hand, an accelerated healing and enhanced TGF-*β* expression have been reported in IFN-*γ* KO mice compared to WT mice [140]. On the other hand, a recent study reported a positive IFN-*γ* contribution to the skin wound healing process, especially in the neutrophilic inflammatory response at the wound site [141]. In fact, IFN-*γ* KO mice displayed significant attenuation in wound closure, wound breaking strength, and myofibroblast differentiation in the proliferation phase compared to WT mice through prolonged neutrophil accumulation and enhanced MMP-2 activation.

**Figure 5:**
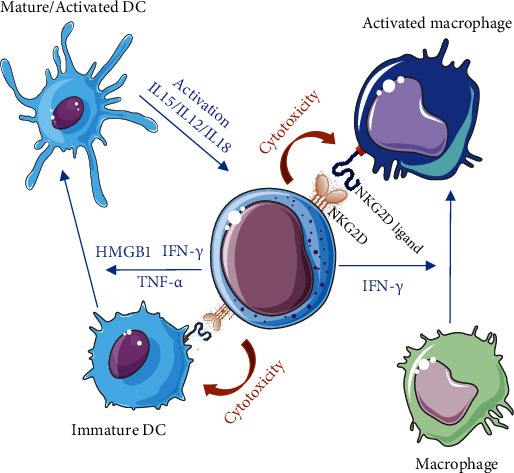
Crosstalk of NK cells with immature DC/activated DC and with resting or activated macrophages either by soluble mediators (blue arrows) or by direct receptor-mediated cell-cell interaction (orange arrows).

The role of NK cells on fibrosis and tissue regeneration could be explained by their double function of cytotoxicity and immunoregulation capacity. They can produce cytokines and growth factors that directly support tissue repair, or they can interact with other cell types, including DCs and macrophages, to indirectly modulate the wound healing process (Figure 5). NK cells promote functional DC maturation and activation via direct cell-cell contact and secreted soluble mediators, including TNF-*α* and IFN-*γ* [142, 143]. In return, IL-18 secreted by activated DC activates the NK cells [21]. Straino et al. reported that the alarmin high-mobility group B1 (HMGB1), a multifunctional proinflammatory cytokine secreted by the NK cells, plays a major role in the maturation of dendritic cells in diabetic wound healing [144].

The interaction of NK cells with macrophages is also a potent first-line defense against pathogens. Indeed, the secretion of IFN-*γ* by activated NK cells contributes to the activation of monocytes into proinflammatory and antimicrobial M1 macrophages [145]. Conversely, macrophages can prime NK cells by two main mechanisms: (1) activation through soluble mediators, such as IL-12 and IL-18, and (2) stimulation through direct cell-cell contact [22, 146]. This contact involves, in particular, the expression of MICA, ULBP1, ULBP2, and ULBP3 at the surface of macrophages exposed to high amounts of LPS. The interaction of these ligands with NKG2D at the surface of NK cells activates these NK to release perforin and granzyme for cytotoxic lysis of overstimulated macrophages [147]. It has also been shown that the release of lytic granules containing perforin and granzymes by NK cells is involved in senescent cell elimination [148], leading to the acceleration of the repair and prevention of fibrosis [149, 150].

Of interest, the activating receptor NKG2D on NK cells seems implicated in their role in wound healing and might be a potential target to ameliorate this process. Indeed, Schenkel et al. showed that the stimulation of NKG2D by the injection of NKG2D-stimulating antibodies into the peritoneal cavity of C57BL/6 mice accelerated wound healing compared to wound healing of mice injected with isotype controls; they also showed that the genetic ablation of NKG2D induced a delayed wound healing process [151]. The active role of NKG2D was also documented in the model of corneal epithelial abrasion, where the blockage of NKG2D receptor on NK cells inhibited corneal nerve regeneration and epithelial healing [152]. Thus, NKG2D engagement might be of interest in tissue regeneration, although this remains to be further investigated.

#### 5.3.2. Invariant Natural Killer T Cell (iNKT)

As reviewed by Kumar et al. [153], a unique and heterogeneous T cell population, invariant natural killer T cell (iNKT), shares some functional and phenotypical characteristics with NK cells and interplays between innate and adaptive immunity. These cells coexpress CD3 and CD56 epitope, produce T helper 1 and T helper 2 cytokines (IFN-*γ* and IL-4), and contain high levels of granzyme B and perforin [23]. iNKT can activate several cell types, including NK cells, macrophages, conventional CD4^+^, CD8^+^ T cells, and B cells, and can also recruit myeloid dendritic cells [22, 154–156]. They infiltrate the cutaneous wounds during the first hours of the inflammatory phase and reach maximal numbers by day 1 until day 3 [157]. The role of iNKT in wound healing is still not fully understood. A genetically modified mouse (J*α*18KO mice deficient in invariant NKT) has permitted to identify a positive contribution of these cells in tissue repair of chronic wounds by avoiding a prolonged inflammatory response mediated by neutrophils; they also activate macrophage phagocytic capacity and their secretion of VEGF, essential for angiogenesis, through the early production of IFN-*γ* [158]. Moreover, they stimulate fibroblasts to produce TGF-*β*, leading to myofibroblast differentiation and increased collagen deposition that favor wound closure [159].

#### 5.3.3. “Non-NK-ILC”: ILC-2

Numerous innate lymphoid cells (ILCs) are present in the healthy dermis at a high abundance as compared to other tissue barriers. There are three groups of ILCs classified according to the transcription factor pathways involved in their differentiation from a common precursor, as well as according to their signature cytokine production and their specific function: (1) the first group of ILCs corresponds to NK cells, also called ILC1s, which express the transcription factor T-bet, with a Th1 cytokine signature; (2) group 2 of ILCs referred to as ILC2s expresses GATA-3 signaling pathway and produces Th2 cytokines; (3) group 3, ILC3, expresses (ROR)*γ*t and is characterized by a Th17 cytokine signature [160, 161]. The most prevalent ILC population within human skin is the ILC2 population, enriched under inflammatory conditions [162]. ILC2s can be mainly activated by predominant epithelial cell-derived alarmins, such as TSLP, IL-25, and IL-33. As introduced, they are characterized by the expression of GATA3 and secretion of Th2-associated cytokines such as IL-5 and IL-13 [163, 164], which may induce eosinophilia, mucus production from goblet cells, activation of M2 macrophages, muscle contractility, mastocytosis, and antihelminthics and allergic immune responses [24]. In particular, in the pathogenesis of cutaneous atopic disease, human ILC2s have been shown to infiltrate the skin after allergen challenge, where they were able to produce Th2 cytokines IL-5 and IL-13 by the IL-33 signaling pathway via the IL-33 receptor ST2 expressed on their surface [165]. ILC2s contribute to tissue repair of various organs, including, as reported in mouse models, the lung following influenza infection [166] or the intestine after intestinal injury [167]. These studies highlighted the major role of ILC2 in tissue injury, notably through the secretion of amphiregulin (AREG), a ligand of the epidermal growth factor receptor (EGFR), in response to epithelial IL-33. The protective role of amphiregulin has also been demonstrated in a mouse model of renal ischemia-reperfusion injury (RIR) by the abolishment of its protective effect following the deletion of AREG in ILC2 using CRISPR-Cas9 [168]. This study also underlined the beneficial role of epithelial IL-33 and ILC2 in reducing RIR mouse mortality. To note, this renoprotection required ILC2 production of amphiregulin and was associated with the presence of M2 macrophages in the kidney; this suggests that the IL-33-ILC2 axis in renal IRI could be potentiated as a therapeutic strategy.

Similarly, in the skin, using a splinted excisional wound mouse model, ILC2s were identified as innate immune cells with an essential role in maintaining tissue integrity [169]. An increase of IL-33 in wound bed was indeed observed between the 3^rd^ and the 5^th^ day postwounding, with an accumulation and an increase in the frequency of ILC2. This work demonstrated that cutaneous injury promotes an IL-33-dependent ILC2 response and that abrogation of this response impairs reepithelialization and wound closure. This study also provided results about an increased ILC2 activation in acute wounds of human skin. Another main role of the activation of ILC2 in response to IL-33 has been shown to involve the polarization of macrophages into the M2 phenotype [170, 171]. In particular, the IL-33 delivery in the diabetic wound has been shown to accelerate wound closure by favoring M2 macrophage polarization *in vitro* and *in vivo* and by increasing fibroblast proliferation under M2 macrophages conditioned medium [172]. Nevertheless, the interplay between macrophages and the innate lymphoid cells might generate fibrosis and favor chronic wound scars, altogether suggesting that the manipulation of IL-33-mediated signal and the modulation of ILC2 activity might be a potential therapeutic approach for skin wounds, providing a basis for improved immunotherapy [173].

## 6. Conclusion

In this review, the contribution of the innate immune system to establishing an effective wound healing was discussed, and the necessity of a fully functioning immune system to create an adequate inflammatory response was highlighted to favor efficient wound closure. The immune system is highly involved in wound reepithelialization, and the delicate immunological balance in the skin deserves further investigation to develop new therapeutic regimens and to get clinical improvement. In the future, better knowledge of novel efficient therapeutic strategies will allow to use immune-stimulating or suppressing molecules to accelerate wound healing.

## Figures and Tables

**Table 1:** Major roles of resident or recruited innate immune cells in the inflammatory phase.

Cells	Major role	Inflammatory mediators	References
Keratinocytes	Primary defense Release of alarmins and AMPs	MCP-1 IL1*β*, GM-CSF, TNF-*α*	[11]
Langerhans cells	Monitoring the presence of infection and damage within the epidermis Antigen-presenting cells (APCs)	IL2 IL-12, IL-23 IL-10	[9]
Dendritic cells	Antigen-presenting cells (APCs) priming naïve T cells	TNF-*α*, CXCL-10, IL-6	[12]
Mast cells	Vasodilation Source of inflammatory mediators Neutrophil recruitment	Histamine Leukotrienes Prostaglandins Proteases Cytokines	[13] [14]
Neutrophils	Phagocytosis and digestion of bacteria, pathogens, and tissue debris	Proteases TNF-*α* IL1-*α* and *β*	[15] [16]
Monocytes/ Macrophages M1 Macrophages M2	Efferocytosis Phagocytosis/secretion of proinflammatory cytokines Secretion of anti-inflammatory cytokines/promote repair Revascularization and wound reepithelialization	TNF/IL-6/IL-1*β* IL10/IL-1RII PDGF/FGF/VEGF, TGF-*β*/TGF-*α*	[17] [18] [19]
NK	Cytotoxic against bacteria, viruses, and senescent cells Immunoregulatory cells	IFN-*γ*, TNF-*α*, IL10	[20] [21] [22]
iNKT	Immunoregulatory cells	IFN-*γ*, IL-4	[23]
ILC2	Activation of macrophage M2	IL-5, IL-13	[24]

## Abbreviations

AMPs: Antimicrobial peptides
APC: Antigen-presenting cell
AREG: Amphiregulin
BMCMCs: Bone marrow-derived cultured MCs
CSF: Colony-stimulating factor
CXCL8: C-X-C motif chemokine ligand 8
CXCR2: C-X-C motif chemokine receptor 2
DAMPs: Damage-associated molecular patterns
DCs: Dendritic cells
ECM: Extracellular matrix
EGFR: Epidermal growth factor receptor
ETs: Extracellular traps
FGF: Fibroblast growth factor
FKDP: Fur keratin-derived powder
GM-CSF: Granulocyte-macrophage colony-stimulating factor
HMGB1: High-mobility group B1
ICAM: Intercellular adhesion molecule
IFN-*γ*: Interferon-*γ*
IP-10: IFN-*γ*-induced protein 10
IL: Interleukin
ILCs: Innate lymphocyte cells
KCs: Keratinocytes
LCs: Langerhans cells
LTB4: Leukotriene B4
MCP-1: Monocyte chemoattractant protein-1
MCs: Mast cells
METosis: Macrophage Extracellular Traps (osis)
MMPs: Matrix metalloproteinases
NETosis: Neutrophil Extracellular Traps (osis)
NK: Natural killer
iNKT: Invariant natural killer T cell
PAMPs: Pathogen-associated molecular patterns
PDGFs: Platelet-derived growth factors
PF4: Platelet factor 4
PRR: Pattern recognition receptor
RIR: Renal ischemia reperfusion
ROS: Reactive oxygen species
TGF-*β*1: Tumor growth factor
TLRs: Toll-like receptors
TNF-*α*: Tumor necrosis factor-alpha
TSLP: Thymic stromal lymphopoietin
VEGF: Vascular endothelial growth factor.
